# Research on Channel Selection and Multi-Feature Fusion of EEG Signals for Mental Fatigue Detection

**DOI:** 10.3390/e23040457

**Published:** 2021-04-13

**Authors:** Quan Liu, Yang Liu, Kun Chen, Lei Wang, Zhilei Li, Qingsong Ai, Li Ma

**Affiliations:** School of Information Engineering, Wuhan University of Technology, Wuhan 430070, China; quanliu@whut.edu.cn (Q.L.); yangliu-cs@whut.edu.cn (Y.L.); wanglei@whut.edu.cn (L.W.); zhileili@whut.edu.cn (Z.L.); qingsongai@whut.edu.cn (Q.A.); excellentmary@whut.edu.cn (L.M.)

**Keywords:** brain fatigue detection, EEG signal, channel selection, sparse representation, feature fusion

## Abstract

With the rapid development of modern social science and technology, the pace of life is getting faster, and brain fatigue has become a sub-health state that seriously affects the normal life of people. Electroencephalogram (EEG) signals reflect changes in the central nervous system. Using EEG signals to assess mental fatigue is a research hotspot in related fields. Most existing fatigue detection methods are time-consuming or don’t achieve satisfactory results due to insufficient features extracted from EEG signals. In this paper, a 2-back task is designed to induce fatigue. The weight value of each channel under a single feature is calculated by ReliefF algorithm. The classification accuracy of each channel under the corresponding features is analyzed. The classification accuracy of each single channel is combined to perform weighted summation to obtain the weight value of each channel. The first half channels sorted in descending order based on the weight value is chosen as the common channels. Multi-features in frequency and time domains are extracted from the common channel data, and the sparse representation method is used to perform feature fusion to obtain sparse fused features. Finally, the SRDA classifier is used to detect the fatigue state. Experimental results show that the proposed methods in our work effectively reduce the number of channels for computation and also improve the mental fatigue detection accuracy.

## 1. Introduction

With rapid economic development, people’s life rhythm is getting faster and faster, and the pressure of competition is becoming greater and greater. High-intensity use of the brain for a long time can easily cause mental fatigue. Mental fatigue has become a sub-health state that seriously affects people’s normal life, which might cause heart disease, palpitations, and other physiological diseases, and may also cause insomnia, anxiety, depression, and other mental diseases [1]. It is very important and necessary to timely detect mental fatigue and make feedback. There are subjective and objective methods for fatigue detection [2]. The subjective detection method mainly evaluates the fatigue state via the subjects filling out the scale based on their subjective mental state and feelings. The commonly used scales include Karolinska sleepiness scale (KSS), Stanford sleepiness scale (SSS), Chalder fatigue scale, et al. [3,4]. The advantage of the subjective evaluation method is ease of use, but the standards are not uniform, and the results are not comprehensive. The objective evaluation method is to obtain the physiological data parameters or biochemical data parameters of the subject under different brain fatigue states through external experimental equipment to evaluate the fatigue state of the subject. According to the information used, objective evaluation methods are divided into psychological and behavioral evaluations, biochemical evaluations, and physiological evaluations. Itoh et al. used sensors to obtain driver’s body movements during driving to assess driver’s fatigue [5]. Fernstrom, et al. conducted related research, and the results showed that the subject’s serum tryptophan will increase as the subject’s fatigue state deepens [6]. Compared with other signals, the electroencephalogram (EEG) signal is considered to be the most accurate and credible assessment of brain fatigue and has been widely recognized by the medical community and scientific research community.

Nowadays, research on brain fatigue detection is not limited to improving the accuracy of fatigue state recognition but also begins to focus on the combination of brain fatigue detection and practical application. This leads to high requirements on the real-time nature of the mental fatigue detection system, the portability of the acquisition equipment, and the complexity of the operation. Considering these factors, it is necessary to reduce the number of brain electrodes to save computation time. Yvonne Trand et al. used 26 channels to obtain EEG data of all subjects before and after fatigue. By analyzing the PSD characteristics and comparing the classification performance of PCA before and after dimensionality reduction, it was found that the results after dimensionality reduction were comparable to the results using the original data [7]. Chen, J et al. used 40 lead channels on the NeuroScan EEG acquisition device to record the EEG signal of the subject in the driving simulation phase on the driving simulator and to study the changes in the mental state of the subject during the simulated driving [8]. Chai, R et al. compared the classification effects of using 32 channels of data and the selected 11 frontal lobe channels (FP1, AF3, F7, F3, FC1, FC2, F4, F8, AF4, FP2, and FZ). The effect of 11-channel data is slightly lower than that of using 32-channel data, but the processing time is reduced [9]. Rifai et al. performed ICA processing on 32-channel data to reduce the channel number to 16. The results showed that the classification effect of the selected 16-channel was comparable to the result using the original 32-channel [10].

In mental fatigue detection based on EEG signals, feature extraction is a crucial step. Whether the extracted features are sensitive to fatigue directly affects the classification performance of fatigue detection. At present, mental fatigue detection methods using EEG signals can be divided into three major categories: power spectrum-based feature analysis, entropy-based feature analysis, and brain network-based feature analysis. Nguyen et al. extracted the power spectrum features of the α-band and β-band, combined with the blink rate, closed-eye rate, and heart rate features to identify the state of the subject. 10 subjects were tested for their proposed method and achieved classification accuracy of 70.5 (±9.9) [11]. Rohit et al. performed spectrum analysis on EEG signals and used support vector machines to predict the state of the 23 subjects. Using EEG spectral features achieved 74% accuracy with SVM and for temporal aggregation of the classifier over a 3 min window improved accuracy to 84% [12]. Wang et al. analyzed the sample entropy value of the EEG signal and combined it with the EMG eigenvalues to establish a mathematical model for fatigue driving evaluation. Combining a PCA-multiple regression model, they got an average accuracy of 95.32% in eight subjects [13]. Zhang et al. calculated the complexity of the approximate entropy of EEG data to distinguish whether the subject had driving experience, combined with the sample entropy characteristics and EOG, EMG signal characteristics to determine the driver’s fatigue level [14]. Han, C et al. applied complex network theory to study the changes of brain network characteristics under different fatigue states. Their results demonstrated under fatigue state, the brain network complexity of 16 subjects improved [15]. Zhao, C used the graph theory method to study the functional network reconstruction changes in different frequency bands of six subjects and compared the brain function network in normal and fatigue states [16]. Only performing feature analysis from a single aspect will cause the loss of effective information. Tzimourta et al. extracted six time domain features and five spectral features for analysis and found that the classification results using two fusion features are more ideal and the best accuracy reached 85.5% [17]. Hu, J. et al. extracted four characteristics of sample entropy, fuzzy entropy, approximate entropy, and power spectrum entropy, and fused together as an input to the gradient-enhanced decision tree to determine whether the driver was in a fatigue state. Under performing on 22 subjects, their results had the average highest accuracy up to 94% [18]. Some closely related methods have been recently developed for EEG based mental health detection. To evaluate the association of EEG with Parkinson’s disease (PD), Peláez Suárez et al., proposed to combine EEG function connection (FC) with graph theory. 30 patients with Parkinson’s disease and 26 healthy subjects were tested. Results demonstrated that PD patients presented alterations in the FC in all frequencies and patients’ neural networks showed less connectivity in the alpha and delta frequencies [19]. Ruiz, Gomez et al. proposed a CCA-based multiplex feature parameters (multiplex clustering coefficient and strength) method to detect Alzheimer’s disease (AD) and showed that the interpretation of the proposed method can be analogous to their frequency-specific counterparts [20].

To solve the problem of complicated calculation induced by channel redundancy when detecting mental fatigue states with multi-channel EEG data, this paper proposes a common channel selection method based on ReliefF algorithm [21]. Considering that the single feature analysis causes the loss of effective information, and multiple feature concatenation causes high dimension of the feature set, this paper suggests a multi-feature fusion method based on sparse representation to improve the classification effect of fatigue state.

## 2. Methods

### 2.1. Channel Selection Based on ReliefF

Relief is a feature selection algorithm by calculating feature weights, mainly for two classification problems [22]. The importance of different features can be expressed by the following formula:

(1) δ=(‖s−M(s)‖−‖s−H(s)‖)

In the above formula, *s* indicates the sample point. M(S) represents the nearest neighbor sample point of the same kind as *s*. In the training data set $S_{1},S_{2},S_{3}\ldots \ldots S_{n}$, sample points $S_{i}(1\leq i\leq n)$ are randomly selected, the nearest neighbor sample point $S_{iNM}$ is calculated as nearest hit in the training data set with the same kind $S_{i}$, and the nearest neighbor sample point $S_{iNM}$ is calculated as nearest miss in the training data set with different classes $S_{i}$. For feature sets F, update the weight values of different features according to the following rules: For a certain feature, if the distance between $S_{i}$ and $S_{iNH}$ is smaller than the distance between $S_{i}$ and $S_{iNM}$, it means that the feature’s contribution to distinguishing the samples of the same type and different types is positive, and the feature weight value should be increased. Otherwise, the feature weight value should be reduced. The complexity of the Relief algorithm is positively related to the number of samples and the number of features. The sampling number is m, the original feature number is a, the threshold of feature weight is δ, the weight of each feature is W, the feature subset is T. The algorithm process is as follows Algorithm 1:
**Algorithm 1.** Relief algorithm
1:**Input:** Features: F, time of iteration: m2:**Initialize** weight value of all features to 0, T is the empty set;3:**For***i* = 1 to m, do4:**Obtain** a sample $S_{i}$ randomly from the training data set S;5:**Calculate**$S_{i}$’s nearest neighbor sample point $S_{iNH}$ from homogeneous training sets, and $S_{i}$’s nearest neighbor sample point $S_{iNM}$ from $S_{i}$ different kinds of training sets;6:**For***j* = 1 to a, do(2)$$W(F_{j})=W(F_{j})-diff(F_{j},S_{i}{}^{F_{j}},S_{iNH}^{F_{j}})/m+diff(F_{j},S_{i}{}^{F_{j}},S_{iNM}^{F_{j}})/m$$7:  **end for**8:**end for**9:**For***j* = 1 to a, do10:**If**$W(F_{j})\geq \delta$11:   **Add** feature $F_{j}$ to T12:**end if**13:**end for**14:**Output:** selected feature subset T

In Equation (2), $F_{j}$ is a certain original feature, and $S_{i}{}^{F_{j}}$ is the value of the sample $S_{i}$ on the feature $F_{j}$. $diff(F_{j},S_{i}{}^{F_{j}},S_{iN}^{F_{j}})$ is defined as follows:

F is discrete:

(3) $$diff(F_{j},S_{i}{}^{F_{j}},S_{iN}^{F_{j}})=\{\begin{array}{cc} 0 & S_{i}{}^{F_{j}}=S_{iN}^{F_{j}}\\ 1 & \mathrm{Others} \end{array}$$

F is continuous:

(4) $$diff(F_{j},S_{i}{}^{F_{j}},S_{iN}^{F_{j}})=\left|S_{i}{}^{F_{j}}-S_{iN}^{F_{j}}\right|$$

The ReliefF algorithm is improved on the basis of the Relief algorithm. The principle of handling multi-classification problems is as follows: Sample points $S_{i}(1\leq i\leq n)$ are randomly selected from the training data set $S_{1},S_{2},S_{3}\ldots \ldots S_{n}$. Calculate the k nearest neighbor sample points of $S_{i}$ nearest hit in the training set of the same kind as $S_{i}$ and record it as $S_{iNH_{h}}^{}(h=1,2..k)$. For different features $F_{j}$, the weight value of each feature is calculated and updated as follows:

(5) $$W(F_{j})=W(F_{j})-\sum_{h=1}^{k}diff(F_{j},S_{i}^{F_{j}},S_{iNH_{h}}^{F_{j}})/(mk)+\sum_{C\notin class(S_{i})}\left[\frac{p(C)}{1-p(class(S_{i}))}\sum_{h=1}^{k}diff(F_{j},S_{i}^{F_{j}},S_{iNM_{h}}^{F_{j}}(C)\right]/(mk)$$

In the above formula, $F_{j}$ is a certain original feature, $W(F_{j})$ is the corresponding weight value, *p*(*C*) is the proportion of a certain category *C* different from $S_{i}$, $p(class(S_{i}))$ is the proportion of the randomly selected sample $S_{i}$ corresponding to the category. $diff(F,S_{1},S_{2})$ is the distance between sample $S_{1}$ and sample $S_{2}$ on feature F. Defined as follows:

F is discrete:

(6) $$diff(F,S_{1},S_{2})=\{\begin{array}{cc} 0 & S_{1}^{F}=S_{2}^{F}\\ 1 & S_{1}^{F}\neq S_{2}^{F} \end{array}$$

F is continuous:

(7) $$diff(F,S_{1},S_{2})=\frac{\left|S_{1}^{F}-S_{2}^{F}\right|}{\max(F)-\min(F)}$$

Suppose the sampling frequency is m, the original feature number is a, the nearest neighbor sample number is k, the feature weight threshold is δ, the weight of each feature is W, and the feature subset is T. The specific steps of the ReliefF algorithm are as follows Algorithm 2:
**Algorithm 2.** ReliefF algorithm1:**Input:** Features: F, time of iteration: m, the number of features: a2:**Initialize** the weight value W corresponding to all features to 0 and T to the empty set;3:**for***i* = 1 to m, do4:**Obtain** the sample point $S_{i}$ randomly from the training data set $S_{1},S_{2},S_{3}\ldots \ldots S_{n}$;5:**Calculate** k nearest neighbor samples $S_{iNH_{h}}^{}(h=1,2\ldots k)$ from the training data set of the same type as $S_{i}$; calculate the k nearest neighbor samples $S_{iNM_{h}}^{}(h=1,2\ldots k)$ from each training data set different from $S_{i}$;6:**For***j* = 1 to a, doEquation (5),7:**end for**8:**end for**9:**For***j* = 1 to a, do10:**If**$W(F_{j})\geq \delta$11:**Add** feature $F_{j}$ to T12:**end if**13:**end for**14:**Output:** selected feature subset T

In this paper, each EEG channel is regarded as a feature, and then the feature selection is carried out according to the above method, so as to select the channel with a high contribution to the classification effect of fatigue EEG data. There are n channels $C_{1},C_{2},C_{3}\ldots C_{n}$ and b features $F_{1},F_{2}\ldots F_{b}$ are extracted from each channel. The weight of channel l can be calculated by the following formula:

(8) $$W(C_{l})=\frac{1}{b}\sum_{i=1}^{b}W(C_{l}^{F_{i}})$$

In the above formula, $W(C_{l}^{F_{i}})$ represents the weight corresponding to the feature *i* on channel *l*, and *b* is the number of features extracted on each channel. The value of $W(C_{l}^{F_{i}})$ can be obtained by the above ReliefF method.

### 2.2. Common Channel Selection Based on Weight Addition

There are m subjects $S_{1},S_{2},S_{3}\ldots \ldots S_{n}$. According to the above method, the channel weight value [23] of each subject is $W_{1},W_{2},W_{3}\ldots W_{m}$. First normalize the channel weights of each subject. After the normalized channel weights are obtained, the normalized weights of the corresponding channels of each test are summed to obtain the common channel weights, as follows:

(9) $$W(C_{i})=\sum_{j=1}^{m}W(C_{i}^{S_{j}})$$

In the above formula, $W(C_{i})$ represents the weight of the *i*-th channel of the last common channel, $W(C_{i}^{S_{j}})$ is the weight of the subject $S_{j}$ channel *i* after normalization. Finally, the weights W are sorted from large to small, and the channel with the highest rank is the common optimal channel combination.

### 2.3. Common Channel Selection Based on Weighted Classification Performance Using a Single Channel

In this paper, the results of each subject’s single-channel performance analysis are combined with the channel weights. The SRDA (Spectral Regression Discriminant Analysis) classifier [24] is used to obtain the single channel classification accuracy rate, and the accuracy rate is weighted to calculate the common channel weight. Suppose there are m subjects $S_{1},S_{2},S_{3}\ldots \ldots S_{m}$ and n channels $C_{1},C_{2},C_{3}\ldots C_{n}$. Extract b features $F_{1},F_{2}\ldots F_{b}$ from each channel, and the calculation method of common channel weights is as follows:

(10) $$W(C_{l})=\sum_{j=1}^{m}\left(\sum_{i=1}^{b}\left(W(C_{l}^{F_{i}}{}_{S_{j}}\right)*Acc(C_{l}^{F_{i}}{}_{S_{j}}))\right)$$

In the above formula, $W(C_{l})$ represents the weight value of the *l*-th common channel, $W(C_{l}^{F_{i}}{}_{S_{j}})$ represents the weight value calculated for the *i*-th feature on the *l*-th channel of the *j*-th subject. $Acc(C_{l}^{F_{i}}{}_{S_{j}})$ represents the classification accuracy rate under the *i*-th feature on the *l*-th channel of the *j*-th subject. Calculate the weight value of each channel according to the above formula, and sort each channel by weight. Finally, select the common channel combination according to the order.

### 2.4. Feature Fusion Based on Sparse Representation

#### 2.4.1. Feature Extraction

Five features in time domain and frequency domain are extracted in this paper. The specific features are shown in Table 1.

#### 2.4.2. Sparse Representation

It is difficult to analyze and process high-dimensional signals, and it is easier and faster to process sparse signals. Sparse signals are signals with few non-zero elements and low dimensions. Most signals are not sparse signals, but in a certain transform domain, the signals can be sparse to obtain a sparse representation of the original signal.

In recent years, the sparse representation theory [25] of signals has attracted the attention of many researchers, a large number of applications are in the field of image signal processing, and there are also a few studies applying sparse representation theory to the classification processing of physiological signals. Sparse representation theory is to represent the original signal with a linear combination of fewer basic signals. Suppose an N-dimensional original signal $X\in R^{N}$, dictionary matrix $D\in R^{N*M}$ containing primitive column signals. Then the signal X can be represented by a linear combination of atoms in D, as follows:

(11) X=DαorX≈Dα,‖X−Dα‖<ε

**Figure 1:**
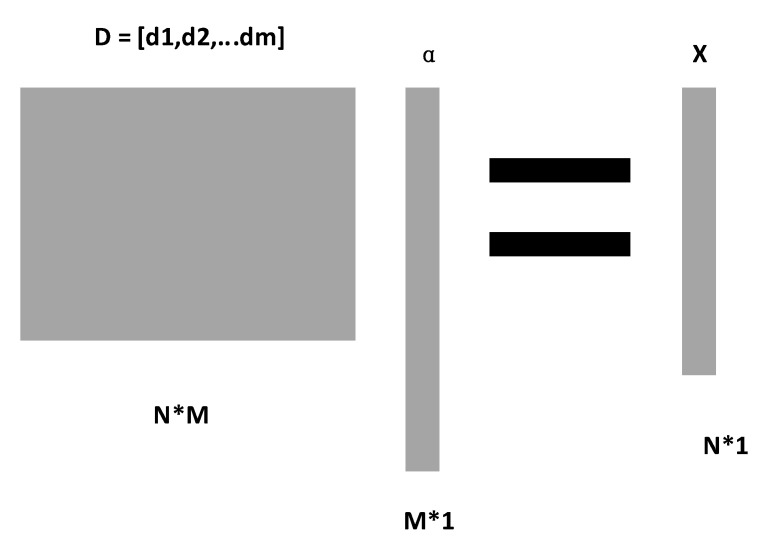
Sparse representation diagram.

In the above formula, $\alpha \in R^{M}={\left[\alpha_{1},\alpha_{2},\ldots ,\alpha_{M}\right]}^{T}$ represents the sparse representation of the original signal X. The sparse representation diagram is shown in Figure 1.

In this paper, in order to reduce the dimension of the feature set, the incomplete dictionary D is used to realize the sparse representation of features.

#### 2.4.3. Multi-Class Feature Fusion Based on K-SVD

The determination of the dictionary in sparse representation theory is a key step. In this paper, K-SVD dictionary learning algorithm [26] is used to design the dictionary matrix.

The K-SVD dictionary learning algorithm is mainly divided into two stages: sparse representation and dictionary update. The sparse representation stage first initializes a dictionary matrix D. Using the OMP algorithm to obtain the sparse representation X of the signal Y is as follows:

(12) $$Y\approx DX=\sum_{i=1}^{M}d_{i}x_{i}$$

The results of the initial dictionary for sparse representation are often not optimal, and dictionary update is required. The dictionary matrix D is updated by columns. First fix the sparse coefficient matrix *X* and dictionary matrix D. Suppose we want to update the *k* column $d_{k}$ of the dictionary matrix, corresponding *k*-th row in the sparse coefficient matrix X is $x_{T}^{k}$.The following formula is available:

(13) $${\left\|Y-DX\right\|}_{F}^{2}={\left\|Y-\sum_{j=1}^{M}d_{j}x_{T}^{j}\right\|}_{F}^{2}={\left\|(Y-\sum_{j\neq k}^{}d_{j}x_{T}^{j})-d_{k}x_{T}^{k}\right\|}_{F}^{2}={\left\|E_{k}-d_{k}x_{T}^{k}\right\|}_{F}^{2}$$

Keep the non-zero value in $x_{T}^{k}$, remove the value of 0, and do the corresponding processing on $E_{k}$, the specific processing is as follows: Define the set $\omega_{k}=\{i|1\leq i\leq N,x_{T}^{k}(i)\neq 0\}$, which represents the set of index values of the signal ${\{y}_{i}\}$ using $d_{k}$. Define $\Omega_{k}$ as a matrix of $N*\left|\omega_{k}\right|$, except that the value at position $\left(\omega_{k}(i),i\right)$ is set to 1, and all other values are 0. Make Equation (13) as follows:

(14) $${\left\|E_{k}\Omega_{k}-d_{k}x_{T}^{k}\Omega_{k}\right\|}_{F}^{2}={\left\|E_{R}^{k}-d_{k}x_{R}^{k}\right\|}_{F}^{2}$$

In the above formula, $E_{R}^{k}=E_{k}\Omega_{k}、Y_{R}^{k}=Y\Omega_{k}、x_{R}^{k}=x_{T}^{k}\Omega_{k}$ respectively show the contraction results of the three after removing 0. Perform SVD decomposition on $E_{R}^{k}$:

(15) $$E_{R}^{k}=U\Delta V^{T}$$

**Figure 2:**
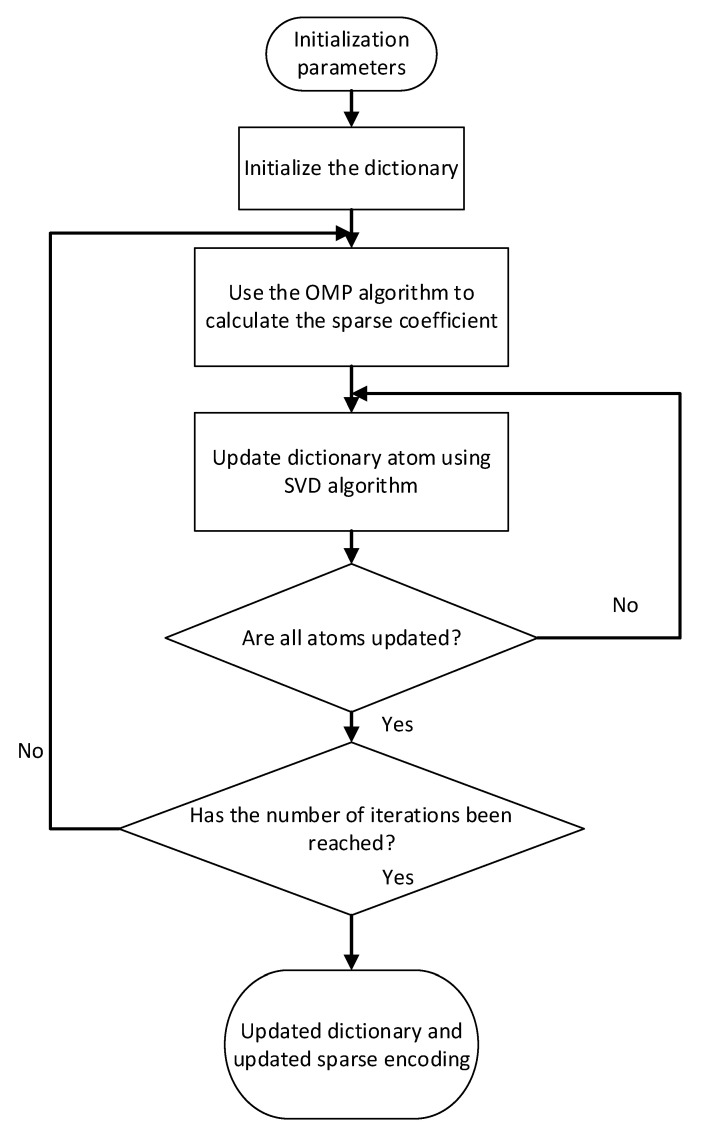
K-SVD algorithm flowchart.

The first column of U is denoted by $\tilde{d_{k}}$, then $\tilde{d_{k}}$ is the update to $d_{k}$. Multiply the first column of V by Δ(1,1) to get the updated result of $x_{R}^{k}$. Follow the above steps to update the dictionary column by column to get a new dictionary $\tilde{D}$, and then use the new dictionary to sparsely represent the original signal. Determine whether the stop condition is reached. If the stop condition is not met, continue to iterate through the above steps until the direct condition is met. The flow of K-SVD dictionary learning algorithm is shown in Figure 2 below.

In this paper, based on the K-SVD dictionary learning algorithm, the dictionary matrix corresponding to the multi-feature set is constructed. Sparse representations of multiple feature sets are used to obtain sparse fusion features, which are used as input to the SRDA classifier to reduce the dimension of the feature set and improve the classification accuracy.

## 3. Experimental Setup

### 3.1. Subject and Experiment Environment

The experimental subjects include eight graduate students without brain disease or other physiological psychological diseases. And they were required not to drink alcohol or take foods, beverages, and drugs that might affect the mental state of the human body during the first 24 h before the experiment. The experiments were conducted in a quiet and closed laboratory, ensuring no external interference, good ventilation, and suitable light. The subjects were asked to sit in a comfortable chair, facing the computer screen, and relax the body to prevent muscle tension from interfering with the results of the experiment. Subjects were instructed and trained about the experimental procedure in advance. And all subjects gave their informed consent for inclusion before they participated in the study. The study was conducted with approval from Wuhan University of Technology.

### 3.2. 2-Back Task

**Figure 3:**
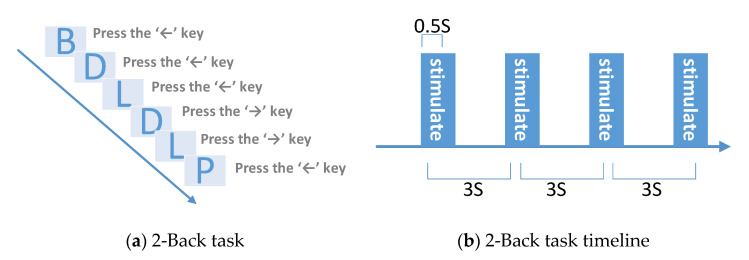
2-Back task stimulation diagram; (**a**) When browsing the stimuli presented sequentially, the subject judges whether the currently presented stimulus matches the N-th stimulus presented before the stimulus; (**b**) For every 3 s was a letter stimulation and the letter presented in the screen for 0.5 s.

The N-back task is a working memory task, which was first proposed by Kirchner and used to examine the age difference in the short-term memory ability of rapidly changing information. In the N-back task, when browsing the stimuli presented sequentially, the subject judges whether the currently presented stimulus matches the N-th stimulus presented before the stimulus. The current stimulation position is marked as M. The subject judges whether it is consistent with the M-1-th stimulation, which is called a 1-back task. The subject judges whether it is consistent with M-2 stimulation, which is a 2-back task. Taking into account the difficulty of the experiment and the experimental experience of the subjects, and studies have shown that 2-back duration of 30 min can induce mental fatigue. In our research, the letter 2-back task is designed to induce mental fatigue. The design diagram is shown in Figure 3.

Subjects need to judge whether the current letter stimulus is consistent with the previous second letter stimulus based on memory. If they are consistent, the subject needs to press the ‘←’ key to respond. If they are not consistent, they need to press the ‘→’ key to respond. When the first and second letter stimuli appear, press the ‘→’ key to respond.

### 3.3. Experimental Design and Procedures

**Figure 4:**
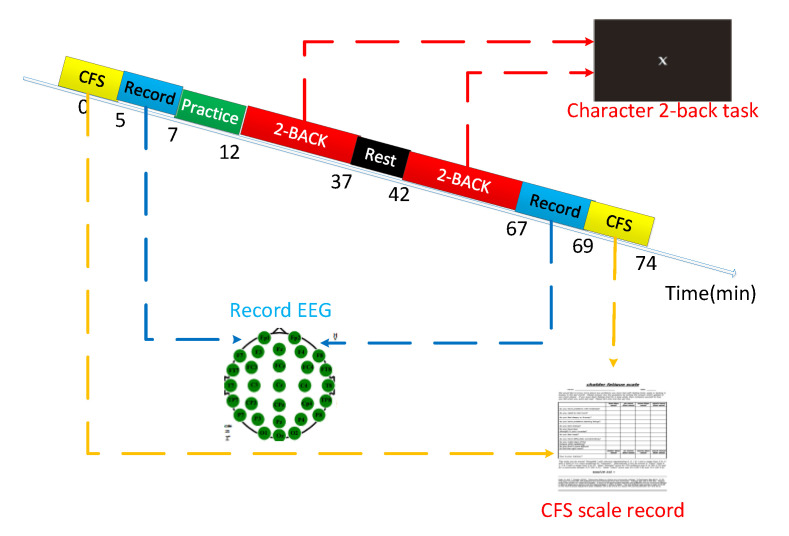
2-Back task induced fatigue experiment design.

The experimental design in this article is shown in Figure 4.

Step 1: The subject put on an electroencephalogram cap and applied conductive paste to sit on the experimental chair and prepare for the experiment.
Step 2: The subject sat and filled in the Chalder fatigue scale.
Step 3: The subject sat quietly on the experimental chair and EEG data were collected for 2 min.
Step 4: The subject began the 2-back task training stage until the accuracy rate reached 0.8 or above.
Step 5: The subject began the first round of 2-back task formal experiment, and the subject’s key response and response time were recorded for 25 min.
Step 6: The subject rested for 5 min.
Step 7: The subject began the second round of the 2-back task formal experiment, and the subject’s key response and response time were recorded for 25 min.
Step 8: The subject sat quietly on the experimental chair and the subject’s EEG data were collected for 2 min.
Step 9: Subject sat and filled in the Chalder fatigue scale.
Step 10: The experiment ended.

**Figure 5:**
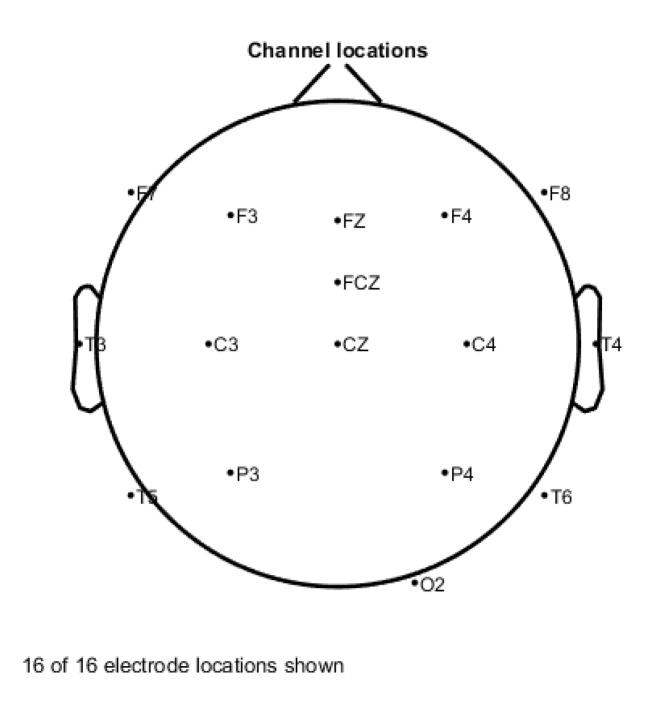
16-channel EEG scalp distribution.

In our research, the following 16 channels are used to collect the fatigue EEG data of the subjects: Cz, FCz, F3, F4, C3, C4, P3, P4, FZ, O, F7, F8, T3, T4, T5, T6. The channel distribution is shown in Figure 5.

## 4. Results and Discussion

### 4.1. 2-Back Task Result Analysis

**Figure 6:**
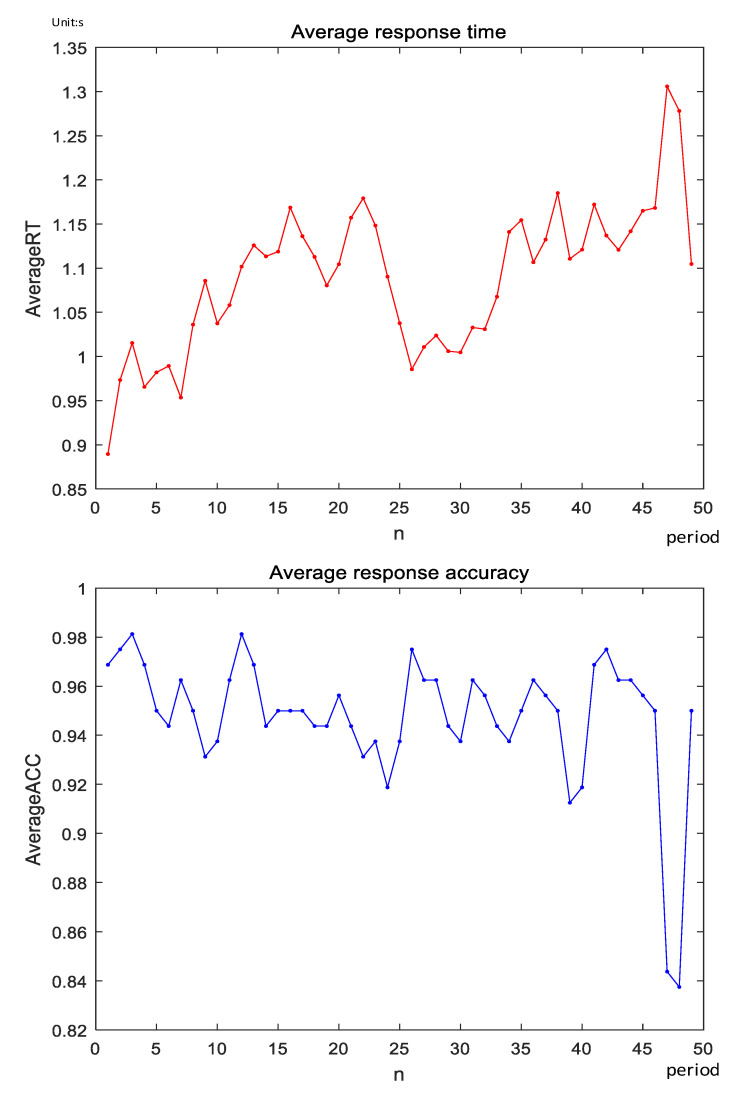
Average response time and average response accuracy of subjects.

In this paper, the reaction time and accuracy of the response to stimulus during the fatigue-induced experiment of eight subjects who completed the 2-back task with a total duration of 50 min were recorded. Average the response time of eight subjects according to the time series to get the average response time in the 50 min 2-back task. Next, the average response time is calculated by a time window with a step length of 2 min and an overlap interval of 1 min. Accuracy of response to eight subjects was treated in the same time window. Finally, the average response accuracy of eight subjects is averaged. The results are shown in Figure 6.

It can be seen from the above figure that although the subjects have a more obvious downward trend around the 25th average reaction time during the completion of the 2-back task. The overall trend is increasing. However, the average response accuracy of the subjects fluctuated less, and only decreased significantly in the final stage of the experiment. The reasons for the above results combined with the feedback analysis after the subject experiment are as follows: The subject gradually experienced fatigue when completing the 2-back task. In order to resist the impact of fatigue, the subject needs to spend a longer time to respond to the stimulus. This subjective resistance ensures that the accuracy rate does not fluctuate much during the 2-back task. The obvious decrease in the reaction time in the 25th period was caused by the subjects entering the second round of task after taking a 5 min break after completing the first round of 2-back task. By the end of the experiment, the subjects were already in a state of high fatigue, which caused a significant drop in accuracy.

### 4.2. Channel Selection Result Analysis

After the 2-back task, the EEG data were recorded. With the 500 Hz sample rate of EEG recorder, 2 min’s data were collected for pre and post fatigue states. Thus, the EEG data size is 16 × 60,000 × 2 of a subject. After segmenting EEG data, the total number of EEG samples was 1264. The datasets were randomly divided into two parts, 75% for training and the rest for testing.

**Figure 7:**
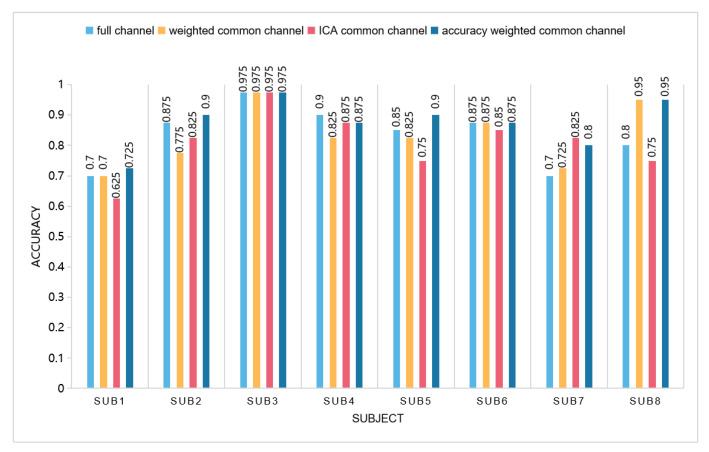
Comparison of classification accuracy of different channel selection methods.

We compare the classification accuracy of eight subjects in the weighted common channel, accuracy weighted common channel, ICA [27] common channel, and full channel. The average accuracy results are shown in Figure 7.

**Figure 8:**
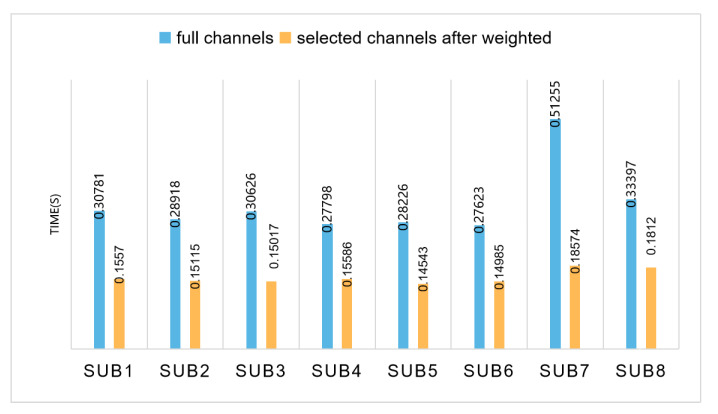
Test time for using full channels and weighted common channels methods.

It can be seen from the figure that, compared with the classification accuracy under the full channel, except that Subject 4 is slightly lower than the classification accuracy under the full channel, the remaining subjects always have one or several channels after selecting basically flat or slightly higher than the classification accuracy under the full channel. Among the three common channel selection methods, the effect of accuracy weighting is overall better than the other two common channel selection methods. While ensuring the classification accuracy, the number of channels is halved, which effectively reduces the complexity of data processing. In addition, the computation time between the methods of full channels and selected channels are shown in Figure 8. It can be seen that the proposed method were less time consuming.

### 4.3. Feature Fusion Result Analysis

**Figure 9:**
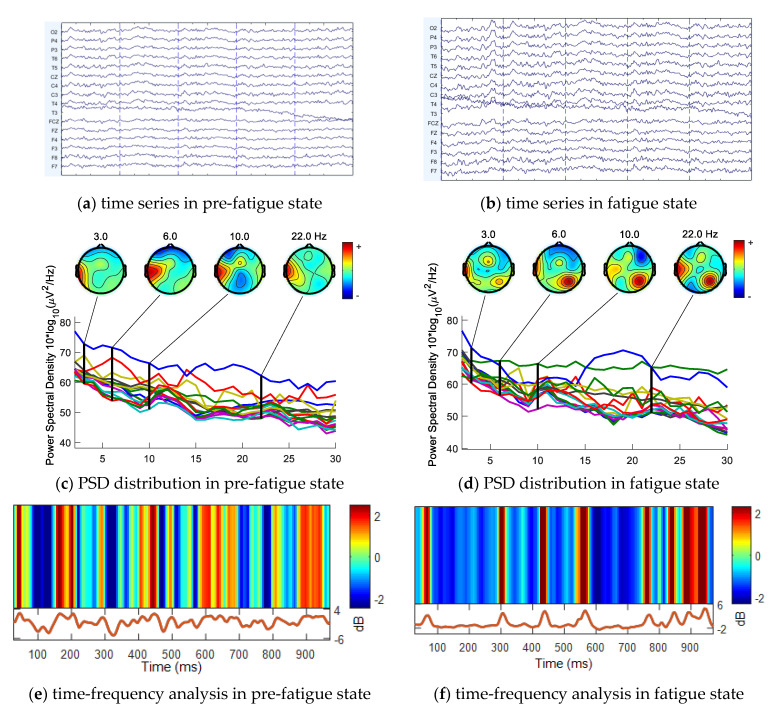
Feature visualization for pre-fatigue and fatigue state. (**a**) 16 channels of EEG time series in pre-fatigue state; (**b**) 16 channels of EEG time series in fatigue state; (**c**) PSD distribution in pre-fatigue state. *X*-axis is frequency from 0–30 Hz and *Y*-axis is PSD value. The lines in the middle are the PSD value of 16 channels from 0–30 Hz. (**d**) PSD distribution in fatigue state. (**e**) Time-frequency analysis in pre-fatigue state. This figure consists of two parts: upper and lower. The upper is the power of frequency in 0–30 Hz and the lower is time series from 0–1000 ms in pre-fatigue state. (**f**) Time-frequency analysis in fatigue state.

Time and frequency domain features are illustrated in Figure 9 plotted with the EEGLAB [28] package in MATLAB.

Figure 9c is the PSD spectrum before fatigue, and the *X*-axis represents the frequency range from 0 to 30 Hz. The four brain topography maps are the energy spectra of the intermediate values 3, 6, 10, and 22 Hz of the four sub bands δ (0.5–4), θ (4–8), α (8–13), and β (13–30), respectively. Before fatigue, the active energy of each sub band was mainly concentrated in the left brain and left temporal lobe, while during fatigue, the energy was mainly distributed in the posterior and right occipital lobe. Under an alert or excited (pre-fatigue) state, it is generally believed that beta waves improve. As the body enters drowsiness or sleep, alpha and delta waves increase in energy. Figure 9e,f are time-frequency graphs of T5 channel within 1000 ms before and after fatigue, respectively. It can be seen that spectrum energy decreases after fatigue compared with pre-fatigue.

In our research, the five extracted single features and sparse fusion features are used as the input of the SRDA classifier. As mentioned above, the total number of EEG samples was 1264. The datasets were randomly divided into two parts, of which 75% were used for training and the rest for testing. The computation was repeated five times. For feature dimension aspect, the dimension of sample entropy and sub-band PSD features is 16 (16 channels*1) for one sample. The dimension of original fused feature is 80 (16 × 5). After K-SVD dictionary learning algorithm, the original dimension was reduced to 20. Classification accuracy of the eight subjects is shown in Table 2.

As can be seen from Table 3, the five extracted features and sparse fusion features are input into the SRDA classifier for classification. Compared with the classification accuracy, the classification effect of the sparse fusion feature obtained by the method in this paper is higher than the classification effect of using a certain feature alone. This shows that the use of multi-class sparse fusion features effectively improves the classification accuracy.

**Figure 10:**
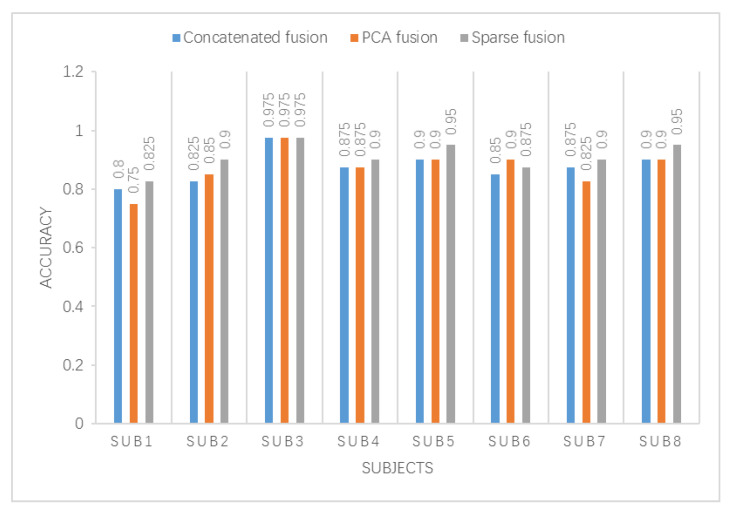
Comparison of fusion feature classification effects.

In addition, the sparse feature fusion method in this paper is compared with the commonly used concatenated feature fusion method and the PCA feature fusion method. The results are shown in Figure 10.

It can be seen from the figure that the three fusion features are used as the input of the SRDA classifier, and the classification accuracy is compared. Subject 6 has the best accuracy of PCA fusion feature classification. Subject 3 has the same effect on the three fusion feature classifications. Subjects 1, 2, 4, 5, 7, and 8 all used sparse fusion features for classification accuracy better than the other two fusion features. The sparse feature fusion method proposed in this paper effectively reduces the dimension of the feature set and improves the classification accuracy.

## 5. Conclusions

Research on brain fatigue detection based on EEG signal is a hot spot in current research. In this paper, a fatigue induction experiment based on the 2-back task is designed, and fatigue EEG data of eight subjects are collected. The channel selection and multi-type feature fusion method of fatigue EEG signal are deeply researched. Based on the ReliefF algorithm, an improved common channel selection algorithm is proposed. Treat each channel as a feature. Use ReliefF algorithm to calculate the weight value of each channel under each single feature, and analyze and calculate the classification accuracy of each channel under each single feature. Finally, the weighted sum of the accuracy is used to obtain the weight value of each channel, and the channel with the highest weight value is the common channel. On this basis, the multi-feature fusion algorithm is further studied. The time domain and frequency domain features are extracted, and multi-class fusion features are obtained as input to the classifier through the sparse representation method.

Experimental results show that the channel selection method proposed in this paper can effectively reduce the 16-channel data to 8 channels while ensuring the classification effect, reducing the amount of data, and reducing the computational complexity. At the same time, the multi-feature sparse fusion method proposed in this paper improves the classification accuracy while ensuring the feature dimension. The brain fatigue detection method based on EEG signal proposed in this paper provides a theoretical basis for the realization of online mental fatigue detection which has important significance.

However, some other problems need to be further researched. The fatigue induction experiment based on the 2-back task in our research is carried out in the laboratory under the shielded environment, which is relatively ideal, and the noise interference of the fatigue EEG data collected is relatively less. How to effectively eliminate the interference of environmental noise and apply mental fatigue detection to the scene closer to real life is worth researching. The fatigue state is a gradual process, and a more detailed quantitative assessment of the fatigue state is needed in the future.

## Figures and Tables

**Table 1:** Feature details.

Features	Feature Details
Frequency domain features	Power spectral density in the beta band (12–30 Hz)
	Power spectral density in the alpha band (8–12 Hz)
	Power spectral density in the theta band (4–8 Hz)
	Power spectral density in the delta band (0.5–4 Hz)
Time domain feature	Sample entropy

**Table 2:** Classification evaluation (mean ± standard deviation) on sparse fusion features method for 5-folds.

Subject	Accuracy	Precision	F1 Score
Sub1	0.825 ± 0.01	0.842 ± 0.071	0.817 ± 0.079
Sub2	0.90 ± 0.04	0.834 ± 0.044	0.897 ± 0.1
Sub3	0.97 ± 0.005	0.962 ± 0.011	0.966 ± 0.04
Sub4	0.90 ± 0.02	0.891 ± 0.05	0.91 ± 0.04
Sub5	0.95 ± 0.10	0.925 ± 0.012	0.906 ± 0.083
Sub6	0.875 ± 0.02	0.84 ± 0.060	0.870 ± 0.08
Sub7	0.90 ± 0.013	0.858 ± 0.121	0.89 ± 0.113
Sub8	0.95 ± 0.025	0.971 ± 0.081	0.959 ± 0.004

**Table 3:** Comparison of average classification effects of different features and sparse fusion features.

Subject	Sample Entropy Feature	PSD (Delta Band)	PSD (Theta Band)	PSD (Alpha Band)	PSD (Beta Band)	Sparse Fusion Feature
Sub1	0.8	0.775	0.65	0.75	0.75	0.825
Sub2	0.9	0.725	0.775	0.725	0.8	0.9
Sub3	0.95	0.95	0.675	0.55	0.7	0.97
Sub4	0.775	0.875	0.85	0.825	0.775	0.9
Sub5	0.875	0.725	0.775	0.925	0.875	0.95
Sub6	0.85	0.575	0.875	0.6	0.8	0.875
Sub7	0.525	0.8	0.625	0.8	0.65	0.9
Sub8	0.925	0.675	0.65	0.775	0.85	0.95

## Data Availability

The data presented in this study is available on request from the corresponding author. The data is not publicly available due to the relevant research still being undergoing.
